# Resections in the Surgical Hospitals and Clinics of Berlin, Prussia

**Published:** 1872-04

**Authors:** A. W. Calhoun

**Affiliations:** Berlin, Prussia


### FOREIGN CORRESPONDENCE.

### RESECTIONS IN THE SURGICAL HOSPITALS AND CLINICS OF BERLIN, PRUSSIA.

BY A. W. CALHOUN, M.D.

During the winter just passed, an unusually large number of resections have been performed in the hospitals of this city, and almost every joint of the body much exposed to disease or injury has undergone this operation, and mostly with exceedingly flattering success. It is a source of remark, that no Semester, during a series of years, has been so prolific as the present in affording both to surgeon and student so much material, and so frequent opportunity of studying this interesting portion of surgery. No special peculiarities in the manner of operating, or in the preliminary or after-treatment, call for any particular notice, for all was of the very simplest character; but the good results attending the large majority of the cases are interesting to note, and serve to diminish our previous fears and doubts as to the favorable prognosis after such surgical interferences.

Trusting the following notes may possess something of interest to many of your readers, I shall give others, pertaining to the same operation, as opportunity permits.

Case I.—Resection of left shoulder-joint. Man aged forty, of a delicate anæmic appearance, but, aside from the diseased shoulder, is apparently quite healthy. One year ago, pain and inflammation began in the joint without assignable cause, and, in spite of almost continual treatment, has increased till the head of the humerus has become a necrosed mass. Two weeks ago, a posterior incision two inches in length was made, and an abscess emptied.

To-day—December 13th, 1871—he again presents himself, and upon thorough examination, the removal of the diseased parts advised. Chloroform is given, and an incision six inches in length is made from a point a little external to the acromio-clavicular joint, directly downward, and to the inner side of the deltoid muscle. After separating the periosteum, together with the attachments of the different muscles surrounding the head and neck of the humerus, shoved out the bone and sawed away one and a half inches, including the head and neck. The slight necrosis of the glenoid cavity was removed by a scraper. Hemorrhage slight, and no vessels requiring ligatures. Passed a drainage tube through the two wounds—the one made to-day, and the one for the relief of the abscess—and closed them in the ordinary way with sutures. Flexing the forearm upon the breast, placed over the limb and left thorax the plaster of Paris bandage, cutting large posterior and anterior windows opposite the wounds, for the purpose of watching and treating them properly without disturbing the position of the arm.

From this time on, the treatment has consisted of local dressings of carbolic acid, twice or three times daily, and a strong nourishing diet. After the second or third week, the suppuration was profuse, and necessitated the removal of the plaster of Paris bandage several times, from its having become saturated with pus. Through the entire progress of the case, the patient suffered comparatively no pain, with but slight fever, but was much reduced by the long-continued suppuration. Two weeks ago—March 15th, 1872—the patient was discharged, having made a good recovery, with free movement of the limb. These movements will, of course, become more perfect and useful when the arm has been more practiced and the parts are less sensitive.

In speaking of the success of this case, Prof. Langenbeck reports that of an officer in whom the head of the humerus was resected about a year ago, and who made so perfect recovery, that to-day he wields his sword in so skillful a manner as scarcely to show any defect in the movements of his arm.

Case II.—Resection of entire humerus, and portion of the radius and ulna at different times, and two successive fractures of the new bone. This case was treated to-day—January 9th, 1872—for second fracture of a new-formed humerus; but as the whole history is unique and quite interesting, I give it *in toto:*

In the battle of Mars-la-Tour, in France, August 16th, 1870, the patient—a Prussian officer, aged twenty-two—received a wound from a Chassepot ball in the right humerus, shattering its head and neck. Owing to the immense number of more severely wounded, his wound remained temporarily dressed till fourteen days after the battle, when resection of the head and upper half of the humerus was performed by the anterior and posterior straight incisions. Wounds healed rapidly, the periosteum depositing new bony substance in an incredibly short time, when every movement of limb and joint was perfect and unimpeded. Five months after this, disease appeared in the elbow-joint and lower half of the humerus, which increased to such an extent, that by the usual operation, the lower or remaining half of the original humerus, together with nearly an inch of the radius and ulna, were removed. He recovered, also, well from this operation, new bone forming and uniting with the upper half, making an entire new humerus, about half the size of the original, and although not so strong, yet quite serviceable, and possessing in the shoulder all the most extensive movements. The elbow is a little stiff. This new humerus has external and internal condyles well-formed, and the upper end of the bone is so rounded and developed as to strikingly resemble the head of the uninjured arm.

In July, 1871, the patient fell and fractured his new humerus at the junction of the upper and middle thirds, which was treated by the plaster of Paris bandage, and firmly healed. The latter part of January, 1872, he again fell, fracturing the same humerus just below the former fracture, which is again treated by a similar bandage, and hyposulphite of lime internally. About the first of March, he presented himself with this second fracture also firmly united, but with considerable atrophy of the muscles of the arm, from long inactivity and and confinement in a plaster of Paris bandage. It is hoped, however, that this will disappear under proper exercise.

In connection with this case, Prof. Langenbeck recited the history of a young man upon whom he operated in 1864, and removed at different intervals the entire humerus, radius, and ulna, with a sound cure, and most of the movements of the arm perfect, the muscles having been left in contact with the periosteum, whereby their action upon the new-formed bones was the same as upon the old.

These are remarkable and instructive cases, showing the rapid formation of bony substance by the periosteum, especially in the young subject, and should make us the more cautious in the excision of any of the bones of the body—to preserve each and every portion of this membrane, uninjured by disease, as its great reproductive powers are here well demonstrated.

Case III.—Resection of elbow-joint. Woman aged forty-eight; married, and has had three children. Was brought into the clinic of Prof. Bærdeleben, January 4th, 1872, with an arm much inflamed and swollen, and several fistulous openings around the elbow, discharging unhealthy pus. The health of the patient has been good previous to ten months ago, when the disease began apparently of its own accord. Examination discovered all the bones composing the joint to be in an advanced state of disease, and, so to speak, *inviting* resection.

Chloroform was administered and one long incision made, posterior to the joint and over the inner edge of the olecranon process, extending from about two and a half inches above to two and a half inches below the elbow, cutting through all the soft parts, and coming immediately upon the bone. Carefully separating the periosteum, with the attachments of the different muscles in contact, opened the joint and turned out the end of each bone, sawing off the diseased part, including the olecranon process of the ulna, cartilage of the radius, and three-fourths of an inch of the articulating surface of the humerus. Any troublesome hemorrhage was prevented by using, instead of sponges, lint saturated in a solution of carbolic acid, which causes the contraction of the smaller blood-vessels, from which the oozing is frequently very annoying. No vessels of consequence required ligation. Having placed in a cloth tent saturated with carbolic acid, for drainage, brought the lips of the wound together by means of sutures; placed the arm, flexed at a little more than a right angle, in a wire splint, and over the whole limb applied the plaster of Paris bandage, cutting a large opening or window opposite the wound.

The patient has progressed to a favorable termination under many unfavorable circumstances. While the wound was so freely suppurating as to reduce her strength to quite a low state, she was attacked with diarrhœa, which came near terminating her life. Recovering from this, erysipelas spread over the whole arm, during the continuance of which the fever was very high. Her vital powers were, however, strong enough to also throw off this complication, and since then, all has gone well. Cleanliness of the parts and carbolic acid dressings (a favorite with Berlin surgeons) have constituted the local treatment. Of course, the above-mentioned complications demanded and received their appropriate management. The plaster bandage was removed several times, but always reapplied as early as possible.

I saw the patient to-day, March 25th, and with the exception of a few granulations over the line of the incision, the wound has healed. There is a marked improvement in general health, strength, etc., and as soon as these few granulations have disappeared, the joint will be exercised in its various proper movements, which it is reasonably supposed the limb will possess.

Case IV.—Resection of the entire carpal bones, metacarpal bones of the index and middle fingers, and a portion of the radius and ulna. Woman aged sixty years; for many years, has suffered from inflammation of the wrist-joint, which, with the adjacent bones, has become so far necrosed that resection is thought advisable. General health very poor, and altogether an unfavorable case for such an operation; but as this is all that promises any benefit, the resection is made, December 1st, 1871. The patient being chloroformed, an incision is made from an inch above the lower end of the radius down along and upon the radial side of the metacarpal bone of the index finger, for its full length, the upper end being afterward extended about half an inch toward the ulna side. Dissecting back the soft parts and periosteum, removed the metacarpal bones of the index and middle fingers, and all the carpal bones, with three-fourths of an inch of the radius and ulna. Introducing a drainage tube, dressed wounds in the usual manner, by bringing the lips together by sutures, and using carbolic acid dressings. The patient lost considerable blood from the ordinarily small vessels, coursing over and around the parts, being immensely enlarged through the long-continued chronic inflammation. The operation, also, was long and tedious, she being under the influence of chloroform for almost an hour.

December 15th.—Patient progressed well for several days, and, notwithstanding all the previous unfavorable symptoms or appearances, hopes were entertained of a good and rapid recovery; but toward the end of a week, some symptoms of pyæmia showed themselves, from which she died a few days thereafter.

BERLIN, PRUSSIA, March 31st, 1872.

